# Stability evaluation of reference genes for gene expression analysis by RT-qPCR in soybean under different conditions

**DOI:** 10.1371/journal.pone.0189405

**Published:** 2017-12-13

**Authors:** Qiao Wan, Shuilian Chen, Zhihui Shan, Zhonglu Yang, Limiao Chen, Chanjuan Zhang, Songli Yuan, Qinnan Hao, Xiaojuan Zhang, Dezhen Qiu, Haifeng Chen, Xinan Zhou

**Affiliations:** Oil Crops Research Institute of the Chinese Academy of Agricultural Sciences, Key Laboratory of Biology and Genetic Improvement of Oil Crops, Ministry of Agriculture, Wuhan, China; Huazhong Agriculture University, CHINA

## Abstract

Real-time quantitative reverse transcription PCR is a sensitive and widely used technique to quantify gene expression. To achieve a reliable result, appropriate reference genes are highly required for normalization of transcripts in different samples. In this study, 9 previously published reference genes (*60S*, *Fbox*, *ELF1A*, *ELF1B*, *ACT11*, *TUA5*, *UBC4*, *G6PD*, *CYP2*) of soybean [*Glycine max* (L.) Merr.] were selected. The expression stability of the 9 genes was evaluated under conditions of biotic stress caused by infection with soybean mosaic virus, nitrogen stress, across different cultivars and developmental stages. ΔCt and geNorm algorithms were used to evaluate and rank the expression stability of the 9 reference genes. Results obtained from two algorithms showed high consistency. Moreover, results of pairwise variation showed that two reference genes were sufficient to normalize the expression levels of target genes under each experimental setting. For virus infection, *ELF1A* and *ELF1B* were the most stable reference genes for accurate normalization. For different developmental stages, *Fbox* and *G6PD* had the highest expression stability between two soybean cultivars (Tanlong No. 1 and Tanlong No. 2). *ELF1B* and *ACT11* were identified as the most stably expressed reference genes both under nitrogen stress and among different cultivars. The results showed that none of the candidate reference genes were uniformly expressed at different conditions, and selecting appropriate reference genes was pivotal for gene expression studies with particular condition and tissue. The most stable combination of genes identified in this study will help to achieve more accurate and reliable results in a wide variety of samples in soybean.

## Introduction

Real-time quantitative reverse transcription PCR (RT-qPCR) is one of the most commonly used techniques to examine transcript levels due to its sensitivity, specificity, wide dynamic range, and high throughput capacity [1–3]. As a valuable tool for basic research, RT-qPCR is available in many fields, such as diagnostics, biotechnology and microbiology [4–9]. However, the accuracy is affected by many factors, such as the quantity and integrity of RNA samples, the efficiency of reverse transcription, PCR amplification and variations in the initial quantities of RNA [3, 10–12]. To avoid the influence of these factors, it is necessary to select ideal reference gene(s) to normalize RT-qPCR analysis. The reliability of the RT-qPCR result depends on carefully chosen experimental operation, and especially on the choice of reference genes to ensure proper normalization [13].

Common reference genes used in RT-qPCR for normalization are housekeeping genes related to basic metabolism pathway, such as *ACTIN* (essential for cytoskeleton structuring and kinetics), *elongation factors* (*ELF1α* and *ELF1β*), *60s* (60s Ribosomal protein L30), *tubulin* (α- and β-tubulin, TUA and TUB, essential for cytoskeleton structuring and kinetic), *Fbox* (Fbox protein family), *Ubiquitin*-conjugating enzyme E2 (family UBC4-enzyme involved in abnormal and short-lived proteins degradation), *glucose-6-phosphate dehydrogenase* (G6PD-important enzyme of the glycolysis pathway) and *cyclophylin* (CYP-central to protein unfolding and protein interaction) [2, 3, 12–15]. As a consensus, it is assumed that these housekeeping genes have constant expression levels among control and treated samples regardless of experimental conditions, developmental stages, tissues and organs or stress treatments [12, 13, 16, 17]. However, a number of studies reported that transcription of housekeeping genes can fluctuate considerably under certain stress conditions, like pathogen infection, cold temperature and drought [18–20]. For example, when maize seeds were infected by fungi, the expression level of some genes involved in metabolism, protein synthesis were down-regulated, including housekeeping genes like *GAPDH* [18, 20]. Thus, there are no universal reference genes under all experimental conditions [17, 21, 22].

During the past few years, there have been lots of reports published with the aim of identifying and evaluating suitable reference genes for expression analysis in different plant species under abiotic stress [11, 13, 16, 19, 23, 24], at different developmental stages or tissues [1, 3, 12, 21, 22], after infection with fungi, virus or bacteria [3, 13, 17, 18, 20, 24, 25]. However, there is a lack of validated reference genes under soybean mosaic virus (SMV) infection and nitrogen stress. Investigating mechanisms of virus resistance and nitrogen stress of soybean have always been our research focus. To understand the expression patterns of genes response to these stresses, RT-qPCR is becoming conventional and has helped decipher the functions of target genes. In this report, we analyzed the expression stability of nine housekeeping genes under different experimental conditions (SMV inoculation, different developmental stages and nitrogen stress) in soybean. To obtain reliable RT-qPCR results, ΔCt approach and geNorm program were both used to evaluate the stability of the nine candidate reference genes. The primary objective of this study was to determine which reference genes demonstrate high stability under specific conditions.

## Materials and methods

### SMV inoculation

The SMV strain SC3 was provided by the National Center for Soybean Improvement (NCSI, Nanjing Agricultural University, Nanjing, China) and maintained in leaves of susceptible cultivar Nannong 1138–2 [26]. The resistant cultivar Zhongdou No.32 (ZD32) and susceptible cultivar Zhongdou No.29 (ZD29) were developed by our institute [27, 28]. These three cultivars were planted in a net house. The SMV inoculums were prepared by grinding leaves of SC3-infected cultivar Nannong 1138–2 to slurry with a pestle in a mortar with moderate 0.01 M sodium phosphate buffer (a mixture of sodium phosphate and potassium phosphate, 5 mL/g leaf tissue, pH 7.2). The inoculation experiments were conducted at V1 stage (Vegetative 1-fully developed leaves at unifoliolate node) of development. The newly expanded unifoliate leaves of the three cultivars were inoculated by rubbing with a paintbrush gently. On the same time, leaves inoculated with 0.01 M sodium phosphate buffer were used as non-infected controls. The inoculated leaves were rinsed with tap water after inoculation. Inoculated leaves of the three cultivars and controls were sampled at 15 min and 6h post SMV inoculation, and frozen in liquid nitrogen and kept at -80°C until RNA isolation.

### Developmental stages

Two soybean varieties Tanlong No. 1 (TL1) and Tanlong No. 2 (TL2) were chosen based their phenotypes. TL1 is characterized by ovate leaflet shape and low seed number per pod and TL2 characterized by narrow leaflet shape and high seed number per pod. They were planted on the farm of our institute with three replications. Each plot contained 10 rows of 3.3 m long, with 0.4 m between rows and 0.1 m between individual plants. Apical buds of the two cultivars were sampled at three soybean developmental stages: Vegetative E (VE-characterized by the presence of the cotyledons), Vegetative 1 and Vegetative 3 (V3-the third node with fully developed leaves).

### Nitrogen stress

Soybean low-N-tolerant soybean variety “Pohuang” (PH) [29] was chosen as the plant material. The mature seeds were germinated and grown hydroponically in one-half-strength modified Hoagland solution containing 2 mM Ca(NO_3_)_2_·4H_2_O, 2.5 mM KNO_3_, 0.5 mM NH_4_NO_3_, 0.5 mM KH_2_PO_4_, 1 mM MgSO_4_·7H_2_O, 0.05 mM Fe-EDTA, 0.005 mM KI, 0.1 mM H_3_BO_3_, 0.1 mM MnSO_4_·H_2_O, 0.03 mM ZnSO_4_·7H_2_O, 0.0001 mM CuSO_4_·5H_2_O, 0.001 mM Na_2_MO_4_·2H_2_O, 0.0001 mM CoCl_2_·6H_2_O [29]. Fourteen-day-old soybean seedlings with cut-off cotyledons were transferred to low nitrogen half Hoagland solutions (10% of normal nitrogen concentration) and high nitrogen (10 times of normal nitrogen concentration) respectively [29, 30]. The culture solution was changed every 3 d. The roots and shoots were sampled separately after 4 h and 6 d of the nitrogen treatment, with three biological replicates per sample. Soybean seedlings in half Hoagland solution without nitrogen treatment were used as controls. The plant tissues were frozen in liquid nitrogen immediately and kept at -80°C until RNA isolation.

### RNA isolation and cDNA synthesis

In all treatments above, samples were collected from 5–10 plants and pooled together. Total RNA was extracted from these samples using TRIZOL reagent (Invitrogen, USA) following the procedures provided by the manufacturer. The quantity of RNA was evaluated by electrophoresis on a 1.2% agarose gel, and the concentration was measured by an Epoch microplate spectrophotometer (BioTek, USA). To eliminate any possible DNA contamination, RNA samples were treated with RNase-free DNase I (Thermo Scientific, USA) according to the manufacturer's instructions. First-stand cDNA was synthesized from 1 μg RNA using the PrimeScript reagent Kit with gDNA Eraser (Takara) in a 20 μL reaction according to the supplier’s protocol. The cDNA samples were stored at -20°C for further analysis.

### RT-qPCR and data analysis

We selected nine candidate reference genes based on previous studies: *60S*, *Fbox*, *ELF1A*, *ELF1B*, *ACT11*, *TUA5*, *UBC4*, *G6PD* and *CYP2* [14, 19, 30, 31], as shown in Table 1. The primer specificity was confirmed by electrophoresis the products of amplification through a 1.2% agarose gel, and all PCR products revealed the presence of the expected amplicons. The quantitative real-time PCR for gene expression was performed on the Bio-Rad thermal using 1×SYBR Green SuperReal Premix (Tiangen, China). Each 20 μL reaction volume contained 4 μL cDNA, 10 μL 1×SYBR Green SuperReal Premix, 4.8 μL dH_2_O and 0.6 μL each primer. The reaction conditions included an initial denaturation step of 95°C for 15 min, followed by 40 cycles at 95°C for 10 s, 60°C for 32 s and 72°C for 30 s with fluorescent signal recording. All experiments were performed in experimental triplicates and biological duplicates. Threshold cycle (CT) data were collected automatically by software supplied with Bio-Rad thermal cycler. Background-corrected raw fluorescence data were exported from the Bio-Rad thermal cycler and analyzed in LinRegPCR software to calculate the amplification efficiency [32]. Mean CT and standard deviations of the 9 candidate reference genes under different stress treatments were calculated (S2 Table) for further analysis.

**Table 1:** List of primer sequence and related information for each candidate reference gene.

Gene	Function	Locus name	Primer sequence	Amplicon length (nt)	Amplification Efficiencies^a^	References
*60S*	60s Ribosomal protein L30	Glyma17g05270	AAAGTGGACCAAGGCATATCGTCG	125	1.868	[19]
			TCAGGACATTCTCCGCAAGATTCC			
*Fbox*	F-box protein family	Glyma12g05510	AGATAGGGAAATTGTGCAGGT	93	1.865	[19]
			CTAATGGCAATTGCAGCTCTC			
*ELF1A*	Eukaryotic elongation factor 1 α	Glyma19g07240	GACCTTCTTCGTTTCTCGCA	195	1.961	[14,19,31]
			CGAACCTCTCAATCACACGC			
*ELF1B*	Eukaryotic elongation factor 1 β	Glyma02g44460	GTTGAAAAGCCAGGGGACA	118	1.933	[14,19,31]
			TCTTACCCCTTGAGCGTGG			
*ACT11*	Actin	Glyma18g52780	ATCTTGACTGAGCGTGGTTATTCC	126	1.924	[30]
			GCTGGTCCTGGCTGTCTCC			
*TUA5*	α-tubulin	Glyma05g29000.1	AGGTCGGAAACT CCTGCTGG	159	1.915	[14,31]
			AAGGTGTTGAAGGCGTCGTG			
*UBC4*	Ubiquitin-conjugating enzyme E2	Glyma18g44850	GAGCGAGCAGTTTCAGAC	168	1.910	[31]
			CATAGGAGGGACGATACG			
*G6PD*	Glucose-6-phosphatedehydrogenase	Glyma19g24250	ACTCCTTGATAC CGTTGTCCAT	126	1.931	[14,31]
			GTTTGTTATCCGCCTACAGCCT			
*CYP2*	Cyclophilin 2	Glyma12g02790	CGGGACCAGTGTGCTTCTTCA	154	1.853	[14,19,31]
			CCCCTCCACTACAAAGGCTCG			

^a^As calculated by LinRegPCR software.

## Results

**Fig 1:**
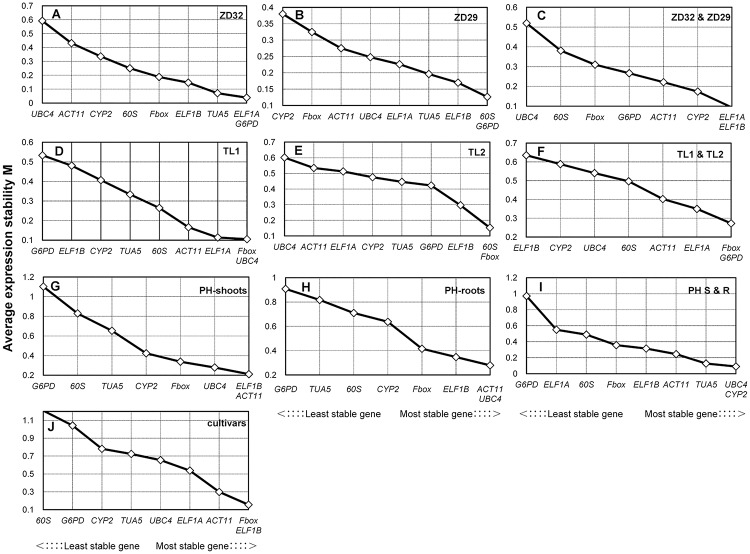
Expression stability and ranking of 9 reference genes under SMV treatment as determined by geNorm. (A) Leaves of ZD32 at 15 min and 6 h post-inoculation with SMV and control. (B) Leaves of ZD29 at 15 min and 6 h post-inoculation with SMV and control. (C) Leaves of ZD32 and ZD29 at 15 min post-inoculation with SMV. (D) Apical buds of TL1 at VE, V1 and V3 stages. (E) Apical buds of TL2 at VE, V1 and V3 stages. (F) Apical buds of TL1 and TL2 at VE and V1 stages. (G) Shoots of PH under nitrogen stress. (H) Roots of PH under nitrogen stress. (I) Shoots and roots of PH under nitrogen stress. (J) Leaves of ZD32 inoculated with SMV, apical buds of TL1 at VE stage and leaves of PH under nitrogen stress.

To obtain high accuracy of stability ranking of the 9 candidate reference genes, two different statistical algorithms ΔCt [33] and geNorm (version 3.5) [34] were used to evaluate expression stability of reference genes. The ΔCt algorithm used the mean of standard deviations of delta Cts to rank the performance of each candidate reference gene [33]. The average standard deviations (STDEV) of the 9 genes were calculated respectively (Table 2 and S3 Table). The lower the values are, the more stable the expression of the reference gene is. For geNorm, the approach determined an average expression stability (M) value for each gene to rank the stable level of the candidate reference genes based on their expression stability [3, 34]. It has been shown that M value and gene stability have a negative correlation [12]. Genes with the highest M value are considered to be the least stable ones, while those with the lowest M value have the most stable expression. The geNorm results for all the experimental sets are presented in Fig 1 and S4 Table.

**Table 2:** Average standard devation (SD) of delta Ct of RT-qPCR.

	SMV inoculation			Developmental stages			Nitrogen stress				cultivars
	ZD32	ZD 29	32^a^ & 29^b^	TL1	TL2	1^c^ & 2^d^		Shoots	Roots	S & R	
***60S***	0.6441	**0.3564**	0.6582	**0.4778**	***0*.*4726***	0.7036		1.2416	0.8713	0.9989	1.7921
***Fbox***	***0*.*4076***	0.5149	**0.4706**	0.5299	***0*.*5275***	***0*.*4742***		**0.8117**	***0*.*8170***	**0.8098**	***0*.*9796***
***ELF1A***	***0*.*4002***	***0*.*3341***	***0*.*3928***	***0*.*4664***	***0*.*5354***	***0*.*5866***		—	—	1.0280	**0.9867**
***ELF1B***	**0.4335**	***0*.*2887***	***0*.*3978***	0.6535	**0.5409**	0.7765		***0*.*7554***	***0*.*7261***	**0.7438**	***0*.*9214***
***ACT11***	0.5526	**0.3433**	—	***0*.*4777***	**0.5779**	**0.6029**		***0*.*8051***	***0*.*8050***	***0*.*6884***	***0*.*8905***
***TUA5***	**0.4519**	0.4193	**0.4104**	**0.5014**	0.6632	—		1.4861	1.1665	***0*.*7037***	1.1903
***UBC4***	1.1844	***0*.*3097***	0.9349	***0*.*4616***	0.8345	0.7755		***0*.*8110***	**0.8638**	—	**1.0901**
***G6PD***	***0*.*4090***	0.3916	0.5097	0.7180	0.5923	***0*.*5127***		1.9231	1.1811	2.4452	1.7061
***CYP2***	0.5418	0.5733	***0*.*3813***	0.5123	0.6667	**0.6497**		**0.9877**	**0.8317**	***0*.*6548***	1.2464

Data obtained for the top five genes are shown in bold letters, while the top three genes are in italic and bold letters.

32^a^, ZD32;

29^b^, ZD29;

1^C^, TL1;

2^d^, TL2.

### Expression stability of reference genes under SMV stress

Since the stability of a reference gene is not constant, the most stable genes in one condition can be highly variable in another. Therefore, we analyzed the data based on individual stresses to search for the best reference gene(s) for each stress treatment. Under SMV treatment, results obtained from ΔCt analysis showed that the top five most stably expressed genes were *ELF1A*, *Fbox*, *G6PD*, *ELF1B* and *TUA5* in resistant cultivar ZD32 (Table 2). When the same data were analyzed by geNorm program, the top five genes were *ELF1A*, *G6PD*, *TUA5*, *ELF1B* and *Fbox* (Fig 1A). Interestingly, the two methods revealed that the top five most stably expressed genes were the same although the exact order was different, and *ELF1A* was the best one identified by two methods. For the susceptible cultivar ZD29, when we compared the data obtained by two algorithms, it turn out that the top five most stably expressed genes were overlapped though the order was not the same (Table 2 and Fig 1B). According to geNorm, *60S* and *G6PD* were the most stable reference genes with a combined M value for both genes of 0.126, while *CYP2* was the least stable reference gene (M = 0.380) in ZD29 infected with SMV (S4 Table). Values of M that surpassed the cutoff value of 0.5 were not considered stable across the treatments [3]. In general, the M values for the majority of the candidate reference genes were below the cutoff of 0.5, with M scores for a few genes above this value. When considering the resistant and susceptible cultivars, *ELF1A* and *ELF1B* behaved best and were the most stable reference genes (M = 0.093) identified by geNorm program (Fig 1c and S4 Table), whereas when using ΔCt method, *CYP2* was the most stable gene with the lowest STDEV value, followed by *ELF1A* and *ELF1B*.

### Expression stability of reference genes at different developmental stages

To confirm the reliability of the potential reference genes at different developmental stages, the validation of the reference genes in TL1 and TL2 was performed. Both the ΔCt method and geNorm algorithm showed similar results, which strengthened the legitimacy of the obtained results. In the developmental series, *UBC4*, *ELF1A* and *ACT11* were the most stable reference genes in TL1 by ΔCt analysis. Similarly, according to geNorm algorithm, *UBC4* and *ELF1A* were still the top ranked reference genes, with M scores of 0.105 and 0.114, respectively [S4 Table]. In TL2 the ΔCt method and geNorm algorithm both ranked *60S*/*Fbox* as the most stable reference gene pair at different developmental stages. When we compared the two cultivars, the top four most stably expressed genes identified by ΔCt and geNorm were *Fbox*, *G6PD*, *ELF1A* and *ACT11*, and even the order were exactly the same (Table 2 and Fig 1D–1F).

### Expression stability of reference genes under nitrogen stress

We next searched for the best reference genes among the 9 selected candidate reference genes for gene expression analysis under nitrogen stress in shoots and roots of PH, respectively. The top five genes in shoots under nitrogen stress were identical via the two algorithms (Table 2 and Fig 1G–1I), of which *ELF1B* and *ACT11* were the most stable genes among all tested genes, while *G6PD* remained to be the least stable one. In roots, *ELF1B* and *ACT11* are the top two stable genes identified by ΔCt algorithm, and they are also included in the top 3 stable genes detected by geNorm software (Table 2, Fig 1H). Finally, when different tissues were considered for stability analysis, ΔCt method showed that the most stably expressed gene was *CYP2*, followed by *ACT11* and *TUA5* under nitrogen stress (Table 2). However, based on the geNorm results, the order of the three best reference genes was as follows: *UBC4*, *CYP2* and *TUA5* (Fig 1I).

### Expression stability of reference genes among three cultivars

The stability of reference genes was dissected in various samples under corresponding stresses, including inoculated leaves of ZD32, apical buds of TL1 at VE stage and shoots of PH under high N stress for 4 h. geNorm results indicated that the most stable genes were *Fbox* and *ELF1B* with a combined M values of 0.155, followed by *ACT11* with 0.299, *ELF1A* with 0.537 and *UBC4* with 0.655 (Fig 1J and S4 Table). When the data were analyzed by ΔCT algorithm, the top five genes were the same (Table 2 and Fig 1J) though the ranking order of expression stability was distinct. The *Fbox* gene was the most stable one when analyzed by geNorm, while it is the third stable reference gene when ΔCt method was used.

### Optimal number of reference genes for normalization

**Fig 2:**
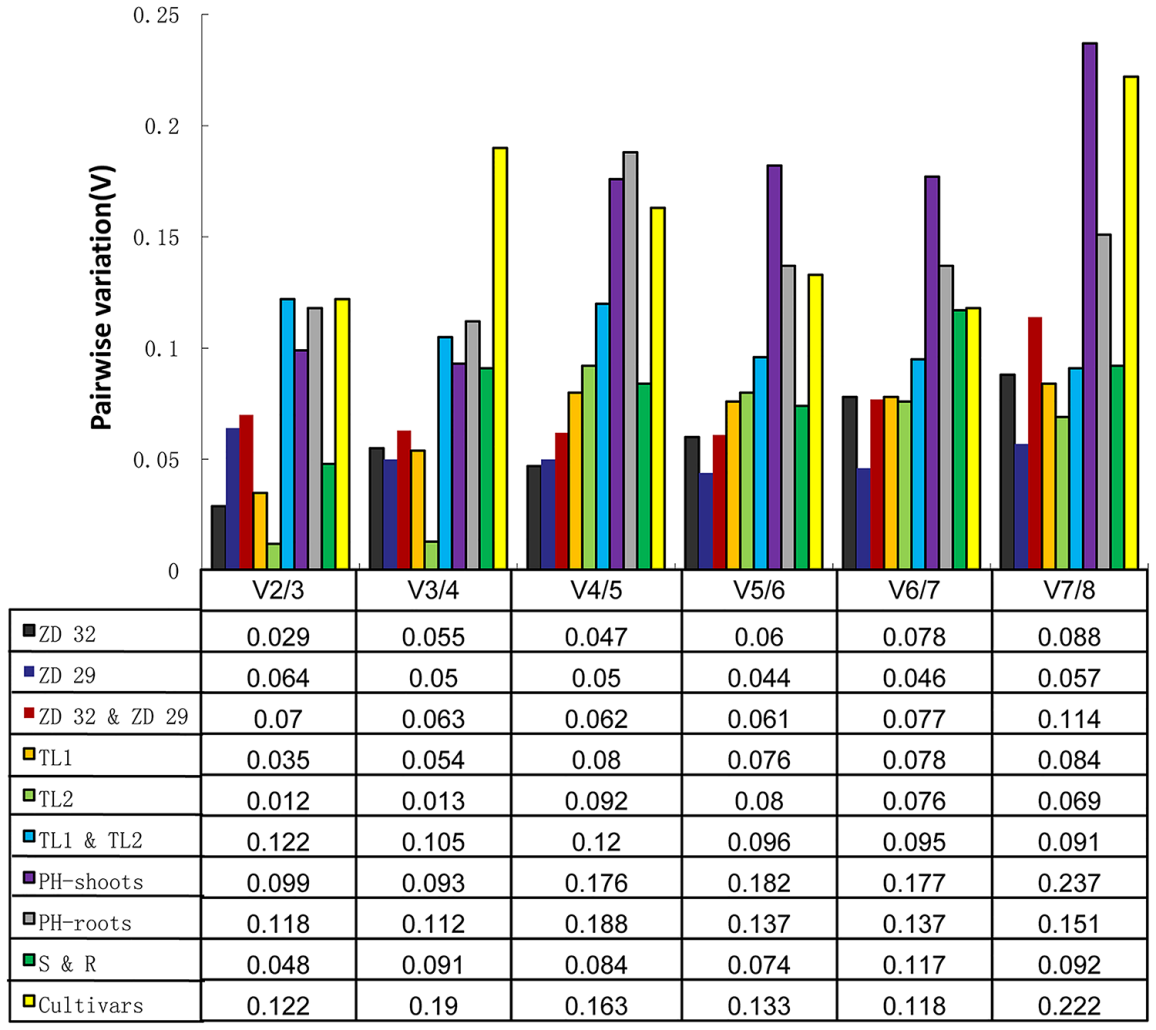
Gene expression pairwise variation (V) of the candidate reference genes calculated by geNorm. The pairwise variation (V_n/n+1_) was analyzed between the normalization factors NF_n_ and NF_n+1_ by geNorm program to determine the optimal number of reference genes required for effective normalization of RT-qPCR data.

The pairwise variations (V_n_/V_n+1_) were also calculated with geNorm between two sequential ranked genes to determine the optimal number of reference genes for normalization under a given set of experimental condition. As suggested by Vandesompele et al. [34], a threshold of 0.15 was set as the cut-off value, below which an additional reference gene was not needed. For example, V_2/3_<0.15 means that the combination of two most stable reference genes was sufficient to normalize the expression of target genes. When considering SMV treatments (ZD32, V_2/3_ = 0.029; ZD29, V_2/3_ = 0.064; ZD32 & ZD29, V_2/3_ = 0.07), different developmental stages (TL1, V_2/3_ = 0.035; TL2, V_2/3_ = 0.012; TL1 & TL2, V_2/3_ = 0.122), nitrogen stress (Shoots, V_2/3_ = 0.099; Roots, V_2/3_ = 0.118; Shoots & Roots, V_2/3_ = 0.048), and different cultivars (ZD 32 & TL1 & PH, V_2/3_ = 0.122) (Fig 2), the V_2/3_ values of all the experimental sets were all lower than the cut-off of 0.15, indicating that it is sufficient to use two reference genes for accurate normalization.

### Recommended reference genes for RT-qPCR in soybean

In present study, two mathematical and statistical models, ΔCt algorithm and geNorm program were used to determine the most suitable reference genes. With comprehensive analyses with geNorm and ΔCt results, we proposed the most suitable gene pairs for normalization of the target genes under specific experimental conditions (Table 3). For example, the pairwise variation V_2/3_ value in TL1 was calculated to be 0.035 by geNorm (S4 Table), suggesting that two most stable genes (*Fbox* and *UBC4*) can be selected for normalization. However, with ΔCt approach, *UBC4* was the most stable gene, *ELF1A* was the second (ranked the third by geNorm) and the *Fbox* gene was the seventh. Therefore, *UBC4* and *ELF1A* would be suitable for RT-qPCR in TL1. Analogous analysis was made for each experimental setting (Table 3). Although we could not identify any single gene expressed constantly under all experimental conditions, one or two appropriate stable reference genes in specific given conditions used in RT-PCR experiments could be recommend. And it should guide the selection of reference genes for gene expression analysis in soybean.

**Table 3:** Recommended reference genes for RT-qPCR of soybean as predicted by geNorm and ΔCt algorithms.

		Recommend genes
SMV stress	ZD32	*ELF1A*/*G6PD*
	ZD29	*ELF1A /ELF1B*
	ZD32 & ZD29	*ELF1A/ELF1B*
		or *CYP2/ ELF1A*
		or *CYP2/ ELF1B*
Developmental stages	TL1	*UBC4/ELF1A*
	TL2	*60S/Fbox*
	TL1 & TL2	*Fbox/G6PD*
Nitrogen stress	Shoots	*ELF1B/ACT11*
	Roots	*ACT11/ELF1B*
	S & R	*CYP2/ACT11*
		or *CYP2/TUA5*
cultivars	ZD 32 & TL1 & PH	*ELF1B/ACT11*

## Discussion

In RT-qPCR analysis, it is assumed that reference genes have constant expression levels among different samples. However, evidences showed that transcripts levels of housekeeping genes may vary considerably under experimental conditions and/or in tissues types [2]. To obtain high accuracy, it is necessary to validate reference genes for each plant species being studied and for each specific experimental condition [3]. The target gene expression was evaluated according to reference gene expression level, thus unstable reference genes can result in inaccurate evaluation of target gene expression. As reported by Dung et al. in the previous study, the expression of four known dehydration-inducible genes *GmNACs* in dehydrate treatment was hard to detect with the least stable reference gene of *SUBI2*, while the relative transcript abundance of *GmNACs* was induced by 3- to 4-fold with the stable reference genes of *60S* and *Fbox* [19]. A previous report also described that when the two most stable genes *GAPDH1* and *EF* were used as reference genes for pistillate flower, the expression level of *AGAMOUS* gene increased gradually in stage 1 to stage 4 and then declined at stage 5. However, when the least stable reference gene *PLA* was used, the expression level of *AGAMOUS* showed fluctuations and failed to achieve a consistent expression pattern [1]. Under salinity or drought stress, the expression of salinity and drought response gene *FaWRKY* in roots of tall fescue peaked at 3 h post salt stress with the most stable reference genes (*SAND* and *TUB*) used, however, the expression of *FaWRKY1* did not show a consistent pattern with the least stable gene (*EF1α*) used [35]. These data indicated that use of suitable internal controls could reveal a more reliable result and is critical for RT-qPCR analysis. Thus, the selection of suitable reference genes in RT-qPCR analysis is pivotal to normalize the transcript expression of target genes.

The algorithms geNorm and ΔCt have been successfully employed to determine the stability of reference gene expression and identify stable reference genes for various plants species [1]. The results obtained from both methods were similar in most of the analyses. For example, *ELF1B* and *ACT11* were found to be the most stable reference genes among different soybean cultivars identified by two ways (Fig 1J, Table 3). In shoots, the top five most stably expressed genes, even their order was exactly the same according to the two methods under nitrogen stress (Fig 1G, Table 2). Some inconsistencies were also found in the ranking order between the two statistical analytical programs, which may be caused by distinct statistical algorithm procedures. In our study, when ZD29 infected with virus, *G6PD* was the best internal control identified by geNorm software, but only ranked the sixth by ΔCt approach and may be inappropriate to normalize in RT-qPCR. Different algorithms strategies may lead to different selection of suitable reference genes, which consistent with previous studies [1, 12, 19, 24, 25]. Thus, the combination of two or more analysis methods to determine the most accurate reference genes for different treatment conditions is necessary.

It has been suggested that the number of reference genes required for quantifying gene expression should depend on the consideration of the research purpose. To get a rough expression of the target gene, one most stable reference gene may be enough, whereas two or more reference genes must be taken if a more accurate expression level was needed [2, 14, 34, 36]. The optimal number of reference genes should be decided based on the threshold of 0.15, nevertheless it is not absolute since small datasets require fewer reference genes than larger ones [12]. In our study, under SMV treatment, the values of V_2/3_–V_7/8_ were totally below 0.15 (Fig 2), meaning 2–7 reference genes were feasible. The results also suggested that V value should only be used as reference, but not the judgment criterion.

The nine commonly used reference genes evaluated here were *60S*, *Fbox*, *ELF1A*, *ELF1B*, *ACT11*, *TUA5*, *UBC4*, *G6PD* and *CYP2*. Similarly to our selected control genes, analogous results were observed in soybean for which *ELF1B*/*60S* and *60S*/*Fbox* were thought to be the most stable gene pairs in roots and shoots respectively under various stresses [19]. In previous studies, we also found that, *ELF1B* exhibited highly stable expression under SMV infection [24] and at different developmental stages [15]. In our study, the gene pairs *ELF1A*/*ELF1B*, CYP2/*ELF1A* or *CYP2/ELF1B* were the most stable reference genes under SMV treatment among resistant and susceptible cultivars. Similar results were obtained by Vívian et al., who reported that *GmCYP2* and *GmELF1A* genes showed relatively stable expression levels in leaves attacked by soybean caterpillar [31]. The expression stability of *CYP* was also described by Bansal et al. [25], who found *CYP* to be a potential stable reference gene during powdery mildew in soybean. It is verified by previous work which showed that *Fbox* was recommended for use in soybean under the conditions of powdery mildew or aphid, and in *Brassica napus* under cold stress or salicylic acid treatment [25, 36]. Similarly, in this study, *Fbox* exhibited stable expression at different developmental stages of TL1 and TL2 (Fig 1D–1F, Table 3).

Although *ACT* is one of the most commonly used reference gene in plants, its expression may vary considerably between tissues and/or samples even within the same plants. We noted that *ACT* has been considered a consistent reference gene and ranked as highly effective for gene expression studies with soybean at different lighting periods [24] and various developmental stages [2]. However, in *Cycas elongate ACT* ranked last indicating low stability across different tissue samples [12]. And the low expression stability was also observed in *Jatropha curcas* [1], *Setaria viridis* [16] and *Nicotiana tabacum* [37]. In present study, *ACT11* was the most stable reference gene in both shoots and roots under nitrogen stress (Fig 1G–1I, Table 3), and it also exhibited stable expression among different cultivars (Fig 1J). While under SMV treatment, the expression of *ACT11* was stable (Fig 1A and 1B) neither in resistant nor in susceptible cultivar. Taken together, these results indicated that suitable reference genes were highly specific for different plant species and particular experimental setups.

We evaluated the independent effect of different experimental sets on the ranking of the reference genes. In the SMV treatment, results indicated that the effect of virus infusion on the expression levels of *ELF1A*, *ELF1B* and *CYP2* was less than that on the other reference genes. These three genes may not be involved in any of the signaling processes of plants in virus defense, and could be considered as reference genes for gene expression analysis in response to virus. For nitrogen stress, *G6PD* was the least stable gene both in root and shoot and should be avoided to be internal control, which was in accordance with the results of other researchers [14, 31, 38], indicating that *G6PD* was not only a component of the glycolytic pathway but also participated in other biological processes.

We noted that *ELF1A* has been considered as the most unstable gene in *Cycas elongate* [12], tomato [18] and tall fescue [35]. While we report the results which are contrary to the previous observations, *ELF1A* appears to be a reliable reference gene under SMV treatment. Our results corroborate those obtained by previous studies, in which *ELF1A* has been considered a stable and effective reference gene in gene expression studies with potato [39], *Populus* [40], poplar [41] and *Caragana* [42, 43]. In addition, we also found that the expression stability of *ELF1A* has shown distinct performance in soybean with different experimental conditions [19, 34, 38]. The contrasting results among different species or within the same species presented a differential expression profile of *ELF1A*, which could be attributed to different treatments. On the other hand, the different results obtained from different studies may be due to the primer pairs that amplify the *ELF1A* member, in accordance with the previous study on the expression stability of 6 soybean *EF1α* genes (named *EF1α1a1*, *EF1α1a2*, *EF1α1b*, *EF1α2a*, *EF1α2b* and *EF1α3*), which was proposed by Saraiva et al. [44]. The third, *ELF1A* may have species diversity in the process of evolution, and its function may have changed among different species. These results provided guidelines for selecting appropriate reference genes in gene expression studies with a particular experimental setting.

## Conclusion

In present study, the expression of 9 candidate reference genes under different experimental conditions was evaluated using ΔCt and geNorm algorithms and the most suitable internal controls for normalizing the data of RT-qPCR under specific conditions in soybean were confirmed.

For SMV infection, *ELF1A*/*G6PD* and *ELF1A*/*ELF1B* should be reliable reference genes for resistant and susceptible cultivars, respectively, and the best reference gene pair is *ELF1A*/*ELF1B* between resistant and susceptible cultivars. For different developmental stages, *UBC4*/*ELF1A* is the best combination for TL1 and *60S*/*Fbox* for TL2. In addition, *Fbox*/*G6PD* should be used as the most suitable reference for comparisons between the two cultivars. For nitrogen stress, *ELF1B*/*ACT11* should be used for the shoots and *ACT11*/*ELF1B* for the roots and *CYP2*/*ACT11* is the best reference gene pair between shoot and root tissues. The combination of *ELF1B* and *ACT11* could be used as the best gene pair for comparison among different cultivars under corresponding stress treatment.

## Supporting information

S1 Table Set of samples (organ/developmental stage/treatment) used for gene expression analysis. (PDF)

S2 Table Average value of CT from three biological replicates±standard deviation (SD) of 9 genes along all treatments. (PDF)

S3 Table Raw data of ΔCt analysis of the expression stability of the candidate reference genes. (XLS)

S4 Table Ranking of candidate reference genes in order of their expression stability as calculated by geNorm. (PDF)
