# Supplementary material for: Stability evaluation of reference genes for gene expression analysis by RT-qPCR in soybean under different conditions
Source: PLoS One. 2017 Dec 13;12(12):e0189405. doi: 10.1371/journal.pone.0189405 (PMC5728501; doi:10.1371/journal.pone.0189405)
Supplement: S1 Table — (PDF) [file pone.0189405.s001.pdf]

S1 Table. Set of samples (organ/developmental stage/treatment) used for gene expression analysis.

No	cultivar	organ	Developmental stage	Time point	Treatment
1	ZD32	Leaf	V1		Control-non-inoculated
2	ZD32	Leaf	V1	15 min	SMV-inoculated leaf
3	ZD32	Leaf	V1	6 h	SMV-inoculated leaf
4	ZD29	Leaf	V1		Control-non-inoculated
5	ZD29	Leaf	V1	15 min	SMV-inoculated leaf
6	ZD29	Leaf	V1	6 h	SMV-inoculated leaf
7	TL1	Leaf	VE		
8	TL1	Leaf	V1		
9	TL1	Leaf	V3		
10	TL2	Leaf	VE		
11	TL2	Leaf	V1		
12	TL2	Leaf	V3		
13	PH	Leaf	V2	4 h	10% normal N concentration
14	PH	Leaf	V3	6 DAI	10% normal N concentration
15	PH	Leaf	V2	4 h	10 times N concentration
16	PH	Leaf	V3	6 DAI	10 times N concentration
17	PH	Root	V2	4 h	10% normal N concentration
18	PH	Root	V3	6 DAI	10% normal N concentration
19	PH	Root	V2	4 h	10 times N concentration
20	PH	Root	V3	6 DAI	10 times N concentration
