# Supplementary material for: Stability evaluation of reference genes for gene expression analysis by RT-qPCR in soybean under different conditions
Source: PLoS One. 2017 Dec 13;12(12):e0189405. doi: 10.1371/journal.pone.0189405 (PMC5728501; doi:10.1371/journal.pone.0189405)
Supplement: S2 Table — (PDF) [file pone.0189405.s002.pdf]

S2 Table. Average value of CT from three biological replicates±standard deviation (SD) of 9 genes along all treatments.

ZD32_SMV	*60S*	*Fbox*	*ELF1A*	*ELF1B*	*ACT11*
			Ct±SD		
Control	21.07±0.316	20.50±0.056	16.84±0.160	21.00±0.127	18.11±0.264
15 min	22.34±0.918	22.66±0.101	18.55±0.098	22.98±0.137	20.90±0.331
6 h	21.19±0.218	21.57±0.111	17.62±0.207	21.70±0.029	20.30±0.147
ZD29_SMV					
Control	21.60±0.223	20.98±0.119	18.05±0.139	21.97±0.085	19.89±0.031
15 min	21.57±0.080	21.84±0.192	17.86±0.070	22.03±0.110	20.16±0.070
6 h	20.89±0.238	21.07±0.082	17.67±0.198	21.52±0.029	19.99±0.044
ZD32 vs ZD29_SMV					
ZD32-Control	21.47±0.206	21.27±0.220	16.95±0.071	21.17±0.278	18.86±0.174
ZD32-15 min	22.74±0.086	22.83±0.109	18.96±0.103	22.98±0.064	21.04±0.284
ZD29-Control	21.72±0.155	22.05±0.042	18.53±0.101	22.55±0.095	20.46±0.209
ZD29-15 min	21.60±0.090	21.88±0.094	18.37±0.143	22.42±0.137	19.78±0.232

ZD32_SMV	*TUA5*	*UBC4*	*G6PD*	*CYP2*
		Ct±SD		
Control	18.87±0.140	18.78±0.399	21.11±0.110	16.08±0.129
15 min	20.38±0.281	22.80±0.107	22.77±0.136	18.79±0.094
6 h	19.56±0.143	21.70±0.147	21.92±0.155	17.66±0.051
ZD29_SMV				
Control	20.30±0.245	21.61±0.032	22.39±0.076	17.05±0.143
15 min	19.88±0.252	21.98±0.210	22.28±0.047	17.64±0.159
6 h	19.35±0.174	21.29±0.113	21.43±0.235	17.69±0.007
ZD32 vs ZD29_SMV				
ZD32-Control		20.05±0.001	21.34±0.181	16.53±0.240
ZD32-15 min		23.83±0.083	23.82±0.145	18.88±0.219
ZD29-Control		22.26±0.152	23.51±0.251	18.07±0.062
ZD29-15 min		22.97±0.113	22.59±0.026	17.86±0.223

TL1	*60S*	*Fbox*	*ELF1A*	*ELF1B*	*ACT11*
Ct±SD					
VE	21.30±0.248	20.35±0.070	17.49±0.100	21.02±0.055	19.28±0.199
V1	20.55±0.093	19.43±0.445	16.83±0.056	21.76±0.126	18.90±0.128
V3	19.61±0.180	19.31±0.052	16.57±0.241	20.06±0.139	18.53±0.166
TL.2					
VE	19.42±0.028	19.27±0.118	16.91±0.191	20.68±0.147	18.82±0.158
V1	19.98±0.073	20.13±0.111	16.84±0.150	21.68±0.047	18.75±0.133
V3	18.95±0.147	18.99±0.081	16.15±0.371	19.88±0.173	18.27±0.104
TL1 vs TL2					
TL1 VE	21.03±0.296	20.10±0.269	17.17±0.179	20.41±0.219	18.86±0.148
TL1 V1	20.31±0.027	19.81±0.024	16.74±0.092	21.50±0.132	18.45±0.135
TL2 VE	19.37±0.184	19.68±0.129	17.21±0.187	20.93±0.022	18.31±0.263
TL2 V1	19.69±0.002	19.68±0.254	17.27±0.110	21.58±0.097	17.74±0.233

TL1	*TUA5*	*UBC4*	*G6PD*	*CYP2*
Ct±SD				
VE	20.41±0.254	20.26±0.261	21.95±0.022	16.95±0.178
V1	19.89±0.241	19.46±0.154	22.50±0.119	17.27±0.174
V3	18.61±0.156	19.13±0.079	20.37±0.296	15.83±0.108
TL2				
VE	19.66±0.270	18.53±0.180	21.38±0.068	17.09±0.339
V1	20.47±0.058	18.71±0.294	21.96±0.077	17.17±0.087
V3	18.13±0.007	18.79±0.109	19.85±0.233	15.24±0.312
TL1 vs TL2				
TL1 VE		21.87±0.039	21.96±0.062	16.87±0.007
TL1 V1		20.69±0.142	22.33±0.125	17.29±0.229
TL2 VE		19.95±0.071	21.87±0.210	17.33±0.107
TL2 V1		20.54±0.168	21.95±0.222	17.72±0.021

PH shoots_N stress	*60S*	*Fbox*	*ELF1A*	*ELF1B*	*ACT11*
Shoot_high N 4 h	25.72±0.152	23.37±0.082		25.32±0.268	22.52±0.119
Shoot_low N 4 h	26.52±0.151	23.67±0.042		25.38±0.032	22.40±0.131
Shoot_high N 6 DAI	26.66±0.222	24.01±0.085		26.03±0.036	23.26±0.071
Shoot_low N 6 DAI	21.93±0.097	20.78±0.175		23.20±0.162	20.73±0.0362
PH roots_N stress					
Root_high N 4 h	29.22±0.243	24.94±0.125		26.19±0.048	24.25±0.069
Root_low N 4 h	29.72±31.73	25.34±0.156		26.21±0.054	23.62±0.156
Root_high N 6 DAI	31.73±0.108	29.73±0.079		30.15±0.050	28.30±0.283
Root_low N 6 DAI	30.48±0.358	26.90±0.067		28.23±0.210	25.66±0.262
PH shoots vs roots_N stress					
Shoot_high N 4 h	27.85±0.168	25.33±0.123	22.01±0.213	26.70±0.034	24.71±0.118
Root_high N 4 h	28.77±0.207	24.59±0.071	22.98±0.067	26.10±0.111	24.32±0.044

PH shoots	*TUA5*	*UBC4*	*G6PD*	*CYP2*
		Ct±SD		
Shoot_high N 4 h	22.18±0.091	23.67±0.101	26.09±0.056	18.10±0.151
Shoot_low N 4 h	21.26±0.119	23.18±0.068	26.73±0.054	18.17±0.148
Shoot_high N 6 DAI	24.23±0.170	23.82±0.070	25.53±0.026	19.04±0.108
Shoot_low N 6 DAI	21.40±0.306	21.05±0.150	20.69±0.013	17.02±0.110
PH roots_N stress				
Root_high N 4 h	24.26±0.351	26.26±0.083	24.78±0.146	20.14±0.287
Root_low N 4 h	24.50±0.263	25.44±0.380	26.29±0.336	20.36±0.217
Root_high N 6 DAI	25.91±0.463	30.08±0.087	30.39±0.163	22.61±0.142
Root_low N 6 DAI	24.56±0.562	28.05±0.264	26.51±0.170	21.21±0.068
PH shoots vs roots_N stress				
Shoot_high N 4 h	23.96±0.061	25.80±0.247	28.69±0.057	19.95±0.103
Root_high N 4 h	24.22±0.123	25.92±0.306	25.30±0.125	19.94±0.184

ZD32 VS TL1 vs PH	*60S*	*Fbox*	*ELF1A*	*ELF1B*	*ACT11*
ZD32 Control	20.09±0.137	19.88±0.056	16.62±0.101	20.37±0.061	17.91±0.097
TL1 VE	21.93±0.124	21.80±0.031	19.12±0.096	22.41±0.085	20.57±0.164
PH shoot_high N 4h	26.17±0.111	23.86±0.084	19.39±0.214	24.16±0.129	21.91±0.089

ZD32 VS TL1 vs PH	*TUA5*	*UBC4*	*G6PD*	*CYP2*
		Ct±SD		
ZD32 Control	18.19±0.104	19.52±0.026	20.11±0.286	14.79±0.008
TL1 VE	20.32±0.278	22.56±0.324	23.69±0.154	18.67±0.145
PH shoot_high N 4h	20.14±0.286	22.29±0.460	26.97±0.171	18.14±0.206
