# Supplementary material for: Stability evaluation of reference genes for gene expression analysis by RT-qPCR in soybean under different conditions
Source: PLoS One. 2017 Dec 13;12(12):e0189405. doi: 10.1371/journal.pone.0189405 (PMC5728501; doi:10.1371/journal.pone.0189405)
Supplement: S4 Table — (PDF) [file pone.0189405.s004.pdf]

S4 Table. Ranking of candidate reference genes in order of their expression stability as calculated by geNorm.

	1	2	3	4	5	6	7	8	9
ZD32	*ELF1A*	*G6PD*	*TUA5*	*ELF1B*	*Fbox*	*60S*	*CYP2*	*ACT11*	*UBC4*
M value	0.039	0.039	0.071	0.147	0.188	0.251	0.336	0.431	0.592
ZD29	*60S*	*G6PD*	*ELF1B*	*TUA5*	*ELF1A*	*UBC4*	*ACT11*	*Fbox*	*CYP2*
M value	0.126	0.126	0.170	0.196	0.226	0.248	0.275	0.325	0.380
ZD32 & ZD29	*ELF1A*	*ELF1B*	*CYP2*	*ACT11*	*G6PD*	*Fbox*	*60S*	*UBC4*	
M value	0.093	0.093	0.174	0.221	0.267	0.310	0.381	0.519	
TL1	*Fbox*	*UBC4*	*ELF1A*	*ACT11*	*60S*	*TUA5*	*CYP2*	*ELF1B*	*G6PD*
M value	0.105	0.105	0.114	0.166	0.264	0.333	0.407	0.480	0.533
TL2	*60S*	*Fbox*	*ELF1B*	*G6PD*	*TUA5*	*CYP2*	*ELF1A*	*ACT11*	*UBC4*
M value	0.153	0.153	0.296	0.423	0.446	0.475	0.512	0.535	0.601
TL1 & TL2	*Fbox*	*G6PD*	*ELF1A*	*ACT11*	*60S*	*UBC4*	*CYP2*	*ELF1B*	
M value	0.273	0.273	0.350	0.402	0.497	0.540	0.588	0.635	
PH-shoots	*ELF1B*	*ACT11*	*UBC4*	*Fbox*	*CYP2*	*TUA5*	*60S*	*G6PD*	
M value	0.211	0.211	0.279	0.338	0.423	0.653	0.829	1.103	
PH-roots	*ACT11*	*UBC4*	*ELF1B*	*Fbox*	*CYP2*	*60S*	*TUA5*	*G6PD*	
M value	0.279	0.279	0.347	0.416	0.637	0.710	0.817	0.908	
PH Shoots & Roots	*UBC4*	*CYP2*	*TUA5*	*ACT11*	*ELF1B*	*Fbox*	*60S*	*ELF1A*	*G6PD*
M value	0.090	0.090	0.127	0.245	0.315	0.357	0.488	0.550	0.971
ZD32& TL1 & PH	*Fbox*	*ELF1B*	*ACT11*	*ELF1A*	*UBC4*	*TUA5*	*CYP2*	*G6PD*	*60S*
M value	0.155	0.155	0.299	0.537	0.655	0.724	0.782	1.042	1.209
